# The role of bariatric and metabolic surgery in the development, diagnosis, and treatment of endometrial cancer

**DOI:** 10.3389/fsurg.2022.943544

**Published:** 2022-08-31

**Authors:** Robert C. Ross, Yetunde M. Akinde, Philip R. Schauer, Carel W. le Roux, Donal Brennan, Amelia M. Jernigan, Marco Bueter, Vance L. Albaugh

**Affiliations:** ^1^ Translational and Integrative Gastrointestinal and Endocrine Research Laboratory, Pennington Biomedical Research Center, Louisiana State University, Baton Rouge, LA, United States; ^2^ Department of Obstetrics and Gynecology, Louisiana State University Health Sciences Center, New Orleans, LA, United States; ^3^ Metamor Institute, Pennington Biomedical Research Center, Louisiana State University, Baton Rouge, LA, United States; ^4^ School of Medicine, St. Vincent's University Hospital and University College Dublin, Dublin, Ireland; ^5^ UCD Gynecological Oncology Group, UCD School of Medicine, Catherine McAuley Research Centre, Mater Misericordiae University Hospital, Belfield, Dublin, Ireland; ^6^ Division of Gynecologic Oncology, Department of Obstetrics and Gynecology, Louisiana State University Health Sciences Center, New Orleans, LA, United States; ^7^ Department of Visceral and Transplantation Surgery, University Hospital of Zürich, Zürich, Switzerland

**Keywords:** bariatric surgery, cancer, obesity, endometrial cancer, metabolic surgery, malignancy

## Abstract

The obesity pandemic continues to contribute to a worsening burden of disease worldwide. The link between obesity and diseases such as diabetes, cardiovascular disease, and cancer has been well established, yet most patients living with obesity remain untreated or undertreated. Metabolic and bariatric surgery is the most effective and durable treatment for obesity, is safe, and may have a protective benefit with respect to cancer incidence. In this review, an overview of the link between obesity, metabolic surgery, and cancer is discussed with emphasis on indications for endometrial cancer, the malignancy most strongly associated with obesity. Considerable evidence from retrospective and prospective cohort studies supports a decreased risk of endometrial cancer in patients with obesity who undergo bariatric surgery compared with nonsurgical controls. Survivors of endometrial cancer are at increased risk of poor health outcomes associated with obesity, and women with endometrial cancer are more likely to die of cardiovascular disease and other obesity-related illnesses than of the malignancy itself. Recent advances in anticancer drug therapies have targeted pathways that may also be therapeutically altered with metabolic surgery. Metabolic surgery has significant potential to enter the treatment paradigm for endometrial cancer, and gynecologic oncologist visits present an opportunity to identify patients who may benefit the most.

## Introduction: obesity, cancer, and bariatric surgery

Obesity, defined by the World Health Organization as excess or abnormal fat that causes a deterioration in health, has surpassed epidemic proportions worldwide and continues to contribute to a worsening burden of diseases like diabetes and cardiovascular disease (1). Aside from its significant financial impact, patients with a BMI >30 kg/m^2^ have been associated with a decreased quality of life and a 25% reduction of disease-free life years (2). Although the link to cardiovascular mortality is long-established, a BMI >30 kg/m^2^ has more recently been linked to cancer (3, 4). Despite this evidence of the association of obesity with cardiovascular disease and cancer, most patients living with obesity remain untreated or undertreated (5).

Metabolic and bariatric surgery is by far the most effective and durable treatment for obesity but remains grossly underutilized. Various benefits of bariatric surgery have been demonstrated in numerous clinical studies that have uniformly identified surgical obesity treatment with increased longevity (6), improved quality of life (7), and decreased cardiovascular morbidity and mortality (8–12). In addition to the studies identifying weight loss in general with cancer-specific benefits (13), bariatric surgery may also have a protective benefit with respect to cancer incidence (14). Thus, recent scientific interest has shifted focus to the potential mechanisms underlying these clinical benefits.

In the following, a general overview of the link between obesity and cancer is reviewed, as well as a focus on the potential for cancer protection following bariatric surgery, specifically with respect to endometrial cancer—the leading malignancy most strongly associated with obesity. We then explore the promise and potential for bariatric surgery to fit into the treatment algorithm for endometrial cancer.

## Obesity and cancer

The link between a BMI >30 kg/m^2^ and cancer is well established. Globally, an estimated 3.6% of all cancers and 12.8% of obesity-related cancers are associated with increasing BMI (15). In general, the effect of obesity on cancer physiology varies depending on the malignancy. As the second and third leading causes of death in the United States, obesity has been implicated as a causal factor in colorectal cancer (16, 17) as well as pancreatic cancer (18, 19). There are 13 types of cancer in the organs strongly related to obesity, namely, esophageal (esophagus), gastric (stomach), colorectal (colon), liver, gallbladder, pancreatic (pancreas), kidney, prostate, breast, ovary, endometrial (endometrium), cervical (cervix), and the hematopoietic system (4). This obesity-cancer link has been demonstrated and confirmed by numerous reviews and meta-analyses, specifically that for every 5 kg/m^2^ increase in BMI >30 kg/m^2^, there is an associated increased risk of developing cancer. This obesity-cancer risk ranges from as low as 9% increased risk for colorectal cancer to as high as a 56% increase of biliary cancers (20). For endometrial cancer, every 10% increase in the waist-to-hip ratio increases endometrial cancer risk by 21% (20). Overall, data continue to mount demonstrating significant proclivity of cancer development in preclinical and clinical obesity studies (21–26).

The overarching question then focuses on “why” obesity is associated with such a proclivity for cancer development. Although there may be unique factors that trigger the development of some malignancies greater than others, general changes associated with obesity and the excess adiposity likely contribute to an increased overall propensity toward cancer growth. Obesity has been described as a chronic inflammatory state, and potentially a state of immunoparalysis (27), which is associated with numerous circulating inflammatory mediators and other growth factors that are elevated relative to a normal weight individual. Insulin, predominately known for its effects on regulating glycemia, is also a strong growth factor proposed to be a driver of carcinogenic pathways. Obesity and insulin resistance are associated with increasing concentrations of circulating insulin that parallel elevated body weight and excess fat mass. This relationship of insulin has been demonstrated in breast cancer studies, as well as other circulating mediators that originate from excess adipose tissue production of other inflammatory mediators (i.e., IL-6, TNF-alpha). Increased obesity and excess adipose tissue provide a feed-forward mechanism that worsens insulin resistance and subsequently further increases insulin secretion from the pancreatic beta cells. This continued worsening of insulin resistance drives excess fatty acid release from adipocytes, as a driver of breast cancer proliferation and migration (28, 29). Increased adipose tissue release of fatty acids is also associated with hepatic insulin resistance and excess hepatic glucose production that can worsen glycemia and drive a further worsening of insulin resistance peripherally (29). Collectively, these inflammatory mediators and the worsening hyperinsulinemia also increase the release of insulin growth factor-1 (IGF-1), a driver of cell growth and proliferation (30), culminating in a milieu of inflammatory and proliferative signals.

## Metabolic and bariatric surgery

**Figure 1:**
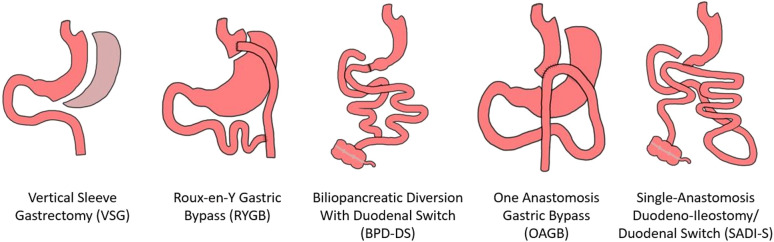
Metabolic/bariatric operations.

Metabolic and bariatric surgery provides the most substantial and durable treatment for obesity (6, 31, 32). Typical patients undergoing metabolic surgery are women (80%) between 35 and 54 years of age (33). In terms of operations (Figure 1), vertical sleeve gastrectomy (VSG) accounts for approximately two-thirds of procedures in the USA. Roux-en-Y gastric bypass (RYGB) accounts for most of the remaining operations, with biliopancreatic diversion with duodenal switch (BPD/DS) making up the remaining primary surgical procedures (34). Bariatric operations successfully treat the disease of obesity through biological mechanisms, many of them remain to be completely elucidated (35–37).

Compared with intensive medical therapy alone, metabolic surgery is associated with more robust weight loss, decreased glycated hemoglobin levels, better glycemic control, decreased requirement for glucose-lowering medications, decreased triglyceride levels, increased HDL cholesterol levels, decreased urinary albumin to creatinine ratio, and an improvement in the quality of life in patients with BMI ≥27 kg/m^2^ (34, 38). A meta-analysis of 17 studies including 174,772 matched participants showed a 49.2% reduction in the hazard rate of death and an increase of 6.1 years of life expectancy in those who underwent metabolic–bariatric surgery compared with usual care (39).

Aside from this remarkable clinical efficacy, metabolic surgery is safe and has a lower 30-day postoperative mortality rate than laparoscopic appendectomy or cholecystectomy (0.3% vs. 0.5% and 0.7%, respectively) and a complication rate similar to that of laparoscopic hysterectomy (3.4% vs. 3.5%) (40). These safety measures have been documented in large retrospective studies (41, 42) and confirmed by prospective clinical trials (43, 44). Even in patients who might be at increased risk secondary to organ failure (heart, lung, kidney failure), the potential benefit of weight loss surgery as a bridge to transplantation has been shown to be remarkably safe and effective (45–48).

## Metabolic surgery and cancer

As the link between obesity and cancer continues to be defined, it remains to be conclusively identified how and to what extent metabolic surgery might modify an individual's cancer risk or even response to cancer treatment. Patients with obesity who undergo bariatric surgery have reduced overall cancer risk compared with controls (49–53), which may be more pronounced in women than in men for reasons not fully understood yet (14). Cancer mortality also decreases, suggesting that metabolic surgery not only protects against developing cancer but also improves patient outcomes after cancer development (52). Individuals who have already developed comorbidities associated with increased cancer risk may also benefit from bariatric surgery. Patients with nonalcoholic fatty liver disease (NAFLD) and a BMI ≥40 kg/m^2^ who undergo bariatric surgery are 16% less likely to develop any cancer and 35% less likely to develop obesity-related cancers than their nonsurgical counterparts (54). In one of the longest studies looking at bariatric surgery and cancer to date with a median follow-up of 21.3 years, Sjoholm et al. reported data from the Swedish Obese Subjects (SOS) study on the effect of bariatric surgery on cancer risk. Incidence of all cancer types was 9.1 per 1,000 person-years in patients who underwent bariatric surgery and 14.1 per 1,000 person-years in the control group (Adjusted HR 0.63, 95% CI 0.43–0.84, *P* = 0.008) (55). The benefits of metabolic surgery on long-term cancer risk are significant, though they remain to be definitively proven through prospective, randomized clinical trials.

Responses to different types of malignancy following bariatric surgery have been increasingly reported as the overall effect of bariatric surgery on cancer development has been identified (Table 1). Although squamous cell carcinoma of the skin has been suggested to have an inverse relationship with obesity and melanoma has had little evidence of any correlation to obesity (56), reported data from the SOS study showed a decreased incidence of both types of skin cancer in patients with obesity who underwent bariatric surgery when compared with nonsurgical matched controls (57). Obesity increases the risk of developing pancreatic cancer, but conclusive data on bariatric surgery patients and ongoing risk reduction are limited (48, 52, 53, 58). Similarly, the relationship of bariatric surgery and colorectal cancer has had conflicting evidence, with some studies demonstrating decreased risk (53, 59, 60), while others showing no change (52, 61) or even a possible increased risk with some but not all procedures (62, 63). Large, prospective clinical trials are necessary to fully identify these effects and whether there may be procedure-specific differences.

**Table 1:** Selected studies examining metabolic/bariatric surgery and cancer response.

Study	Year	Study type	Mean follow-up (years)	Surgery		Control		HR or OR	95% CI	Cancer type
				Events	Total	Events	Total			
Christou	2008	Retrospective cohort	5	21	1,035	487	5,746	0.22	0.14–0.35	Any
McCawley	2009	Retrospective cohort	──	53	1,482	203	3,495	0.62	0.45–0.84	Any
Adams	2009	Retrospective cohort	12.5	254	6,596	477	9,442	0.76	0.65–0.89	Any
Sjostrom	2009	Prospective cohort	10.9	117	2,006	169	2,036	0.67	0.53–0.85	Any
Buchwald	2010	Retrospective cohort	25	38	421	46	414	0.81	0.52–1.27	Any cancer mortality
Ostlund	2010	Retrospective cohort	9	296	13,123	^a^	^a^	^a^	^a^	Any
Derogar	2013	Retrospective cohort	7 control, 10 surgery	70	15,095	373	62,016	^b^	^b^	Colorectal
Ward	2014	Retrospective cohort	──	408	100,000	1,409	100,000	0.29	0.26–0.32	Uterine
Douglas	2015	Retrospective cohort	3.4	130	3,603	142	3,640	0.91	0.72–1.16	Any
Davidson	2016	Retrospective cohort	7.2	31	7,925	75	7,925	0.4	0.25–0.64	Any cancer mortality
Gribsholt	2017	Retrospective cohort	4.2	17	9,895	983	247,366	0.43	0.27–0.70	Any
Maret-Ouda	2017	Retrospective cohort	3.5 control, 3.7 surgery	8	34,437	53	123,695	0.9	0.4–1.9	Esophageal adenocarcinoma
Anveden	2017	Prospective cohort	18.1	97	1,420	135	1,447	0.71	0.59–0.85	Female-specific cancers
Njei	2018	Cross-sectional	──	<10	230,956	18	230,956	0.11	0.03–0.48	Hepatocellular
Mackenzie	2018	Retrospective cohort	4.6	89	8,794	350	8,794	0.23	0.18–0.30	Endometrial, breast, prostate
Pontiroli	2018	Retrospective cohort	17	10	385	35	681	0.49	0.24–1.01	All
Ceriani	2019	Retrospective cohort	──	7	472	96	1,405	0.21	0.09–0.45	Any cancer mortality
Hassinger	2019	Retrospective cohort	5.7	17	2,430	32	2,430	0.53	0.29–0.95	Breast
Kauppila	2019	Retrospective cohort	──	422	49,977	13,880	494,842	0.84	0.76–0.93	Any cancer mortality
Liakopoulos	2019	Retrospective cohort	4.5	153^c^	5,321	188^c^	5,321	0.78	0.63–0.97	Any cancer mortality
Schauer	2019	Retrospective cohort	3.5	488	22,198	2,055	66,427	0.59	0.36–0.97	Any
Tsui	2020	Retrospective cohort	──	1,448	71,000	7,695	323,197	0.87	0.82–0.92	Any
Feigelson	2020	Retrospective cohort	3.4 control, 4 surgery	133	17,998	567	53,889	0.63	0.52–0.76	Breast
Tao	2020	Retrospective cohort	──	1,314	49,096	24,565	433,476	0.89	0.83–0.94	Any
Taube	2020	Prospective cohort	18.1	23	2,007	45	2,040	0.59	0.35–0.99	Melanoma and squamous cell
Tsui	2021	Retrospective cohort	──	1,164	55,781	6,648	247,102	0.78	0.73–0.83	Female-specific cancers
Rustgi	2021	Retrospective cohort	1.9	925	33,435	1,898	64,655	0.84	0.77–0.91	Any
Sjoholm	2022	Prospective cohort	21.3	68	393	74	308	0.63	0.43–0.84	Any
Aminian	2022	Retrospective cohort	6.1	96	5,053	780	25,265	0.68	0.53–0.87	Obesity related

^a^ Surgical cohort compared with background population data, Standardized Incidence Ratio (SIR) = 0.98 (95% CI 0.90–1.07).

^b^ SIR = 1.60 (95% CI 1.25–2.02) in a surgical cohort, 1.26 (95% CI 1.14–1.40) in a nonsurgical cohort.

^c^ Per 10,000 person-years.

Above all other cancers, endometrial cancer has the strongest association with obesity, and data suggest that significant weight loss may even lead to a regression of premalignant pathology (64–66). A decreased incidence of endometrial, breast, and prostate cancer (hormonally responsive cancers) has been associated with RYGB, gastric banding, and VSG (63). The largest reduction in the risk of hormone-related cancers, however, is seen in gastric bypass, suggesting a possible alternative mechanism or variable effect on an underlying mechanism of risk reduction among different types of bariatric surgery (63). A decreased risk of breast cancer has been independently associated with patients who have undergone bariatric surgery compared with nonsurgical controls (52, 67–69). Bariatric surgery has also been associated with a 53% decreased risk of ovarian cancer in women living with obesity (67).

Considerable evidence from retrospective and prospective cohort studies supports a decreased risk of endometrial cancer in patients with obesity who undergo bariatric surgery compared with nonsurgical matched controls (52, 53, 67, 70–72). A prospective pilot study of the effects of bariatric surgery on the risk of endometrial pathology in women undergoing laparoscopic RYGB identified a 6.8% preoperative prevalence of occult hyperplastic endometrium, considered a precursor lesion for endometrial cancer, upon biopsy. At one-year follow-up, half of the patients with identified endometrial hyperplasia had resolution (66).

Information about bariatric or metabolic surgery and cancer has primarily been confined to retrospective or prospective cohort studies. Although attempts have been made to parse out cancer outcomes after bariatric surgery from randomized controlled trials, the relative rarity of these studies, combined with small sample sizes and short follow-up periods, has limited their usefulness (73). Thus, a better understanding of endometrial and other hormonally sensitive cancers, including breast and prostate, is needed.

## Bariatric surgery as a component of endometrial cancer survivorship

More than half of women diagnosed with endometrial cancer in the United States have a BMI >25 kg/m^2^ (74). It is not surprising, therefore, that endometrial cancer survivors are at risk for experiencing poor health outcomes such as type 2 diabetes mellitus, dyslipidemia, obstructive sleep apnea, and cardiovascular morbidity and mortality (75). In women who fail nonsurgical weight loss approaches, bariatric surgery has been shown to reduce the incidence of obesity-related disease and long-term all-cause mortality (76). Because of the excellent cancer-specific outcomes and preponderance of obesity-related complications, women with endometrial cancer are more likely to die of cardiovascular disease and other obesity-related illnesses than endometrial cancer itself (77). This makes an endometrial cancer diagnosis a critical teachable moment and emphasizes the importance of actively managing the underlying issue of obesity in the endometrial cancer survivorship period.

The endometrial cancer survivorship period begins at the time of diagnosis and includes cancer treatment, management of chronic or intermittent disease, and addressing end-of-life care as needed. During this period, patients with endometrial cancer develop close relationships with their gynecologist oncologists. Therefore, it is an opportune time to address approaches to weight loss as patients typically see their oncologist on average at least every 3–12 months. Endometrial cancer survivors report that it is acceptable for their gynecologic oncologist to discuss weight loss strategies (75). However, there are gaps in gynecologic oncologists’ practice patterns as it relates to weight loss counseling. Only 60% of gynecologic oncologists report feeling adequately prepared to provide weight loss counseling and only 50% actually provide it (78, 79). Furthermore, only 10% of gynecologic oncologists report receiving formal training in obesity management (78, 79). Importantly, women are more likely to accept a bariatric referral when it is offered early in the course of endometrial cancer survivorship (75). Thus, gynecologic oncologist visits offer an opportune juncture for interventions focused on correction of metabolic disease, and bariatric referrals are most likely to be acted upon when offered early in the course of cancer care and treatment.

## Bariatric surgery and endometrial cancer treatment

As noted above, obesity plays a crucial role in endometrial carcinogenesis, and an effective intervention has tremendous potential for improving the overall health of endometrial cancer survivors living with obesity. The standard treatment for most early-stage endometrial cancers is hysterectomy, using minimally invasive approaches when possible. This is followed by adjuvant radiotherapy and/or chemotherapy as needed. Unlike women who have healthy weight, women with obesity experience significant intraoperative challenges such as difficulty with optimal positioning, abdominal insufflation, initial abdominal access, and tolerating steep positioning (80). Thus, women with obesity are less likely to undergo minimally invasive approaches and are at a much higher risk for developing postoperative complications (e.g., wound infection, venous thromboses, and prolonged length of stay (81)). As obesity rates continue to rise and childbearing is increasingly deferred, growing numbers of endometrial cancer may be found in women desiring uterine preservation and progestin based therapies may be appropriate (82). Bariatric surgery is effective in achieving weight loss, restoring the hypothalamic–pituitary axis which is deranged in many of these women, and optimizing pregnancy outcomes (83). With the ability to use conservative measures, like the levonorgestrel-releasing intrauterine device (IUD) to buy time, there is tremendous interest in utilizing bariatric surgery to help women lose weight and reach their goals, whether that be candidacy for a safer minimally invasive hysterectomy or optimizing their chances at fertility and healthy pregnancy outcomes.

There is tremendous enthusiasm for employing bariatric surgery early during cancer care. This has been demonstrated to be feasible as a component of delayed hysterectomy as women either pursue pregnancy or weight loss to optimize themselves for a minimally invasive hysterectomy (27). In one case, an adolescent patient treated with the levonorgestrel-releasing IUD for fertility preservation, bariatric surgery with sleeve gastrectomy was employed and she subsequently reached normal body weight and showed complete response to the IUD (84). Another patient with obesity underwent bariatric surgery to become eligible for hysterectomy after failing hormonal treatment with the levonorgestrel-releasing IUD. She successfully lost ∼18% of her total body weight within 8 weeks of her laparoscopic sleeve gastrectomy and was able to have an uncomplicated hysterectomy and bilateral salpingo-oophorectomy for stage IA grade 1 endometrial cancer (85). In a compelling series by Dyck et al., 6/9 women with endometrial cancer treated with VSG experienced a regression of the tumor, suggesting that there may be a therapeutic benefit to surgical weight loss (27). Concurrent Roux-en-Y with a robotic hysterectomy, bilateral salpingo-oophorectomy, and bilateral sentinel lymph node biopsies in the setting of early-stage endometrial cancer treatment has been reported; the woman experienced a subsequent 30% total weight loss as well as reduction in antihypertensive and antidiabetic medication requirements (86). Coordination of these cases is complex and requires both a multidisciplinary team and a well-counseled and well-informed patient. Criteria for optimal patient selection and long-term outcomes in larger patient cohorts remain to be described.

## Bariatric surgery and novel endometrial cancer therapies

Tremendous strides have been made in recent years with regard to the treatment of advanced and recurrent endometrial cancers, with new drug approvals and therapeutic approaches in the immunotherapy, targeted therapy, and hormonal therapy spaces. While enthusiasm for bariatric surgery is likely to be limited to women with early-stage disease with expected excellent cancer-specific outcomes, this wealth of new information in the advanced disease space offers the opportunity to refine our approach to early-stage endometrial cancer. Many novel therapeutics work on the same pathways that are altered with obesity and surgical weight loss, which presents the possibility of pivoting off these advances to improve an understanding of how bariatric surgery might benefit women with endometrial cancer.

**Figure 2:**
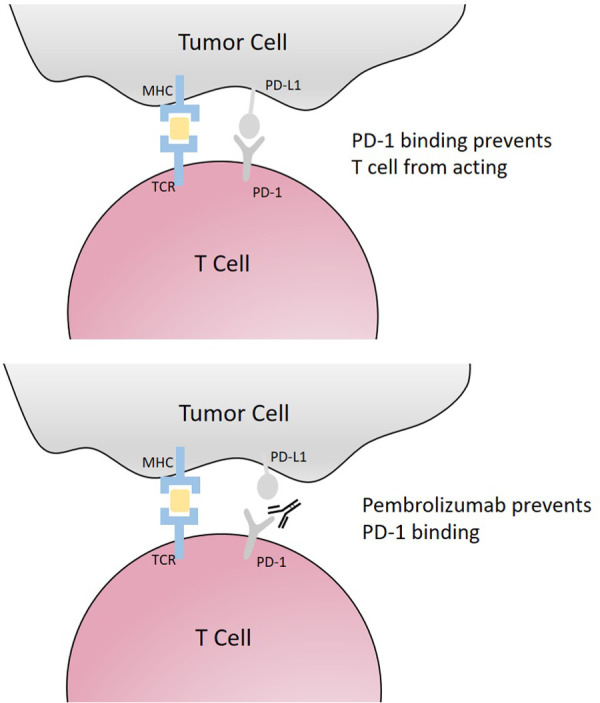
Pembrolizumab mechanism of action. Pembrolizumab prevents PD-1 binding and blocking T-cell and tumor cell interaction, allowing for immune-mediated tumor cell destruction.

KEYNOTE 158 showed a robust and durable response to pembrolizumab for advanced mismatch repair deficient or microsatellite instable endometrial cancer, and the FDA has subsequently granted approval for treatment in this setting (87, 88). Pembrolizumab is a humanized monoclonal antibody that binds PD1 on T cells. Ligands on tumor cells interact with PD1 to downregulate the immune response to the tumor, but with immune checkpoint inhibition, this interaction is blocked, resulting in T-cell recognition of the tumor cell as “foreign” and subsequent antitumor immune response (Figure 2) (89). Importantly, of the four molecular types of endometrial cancers (POLE, mismatch repair deficient, p53 abnormal, or no specific molecular profile), most obese women are likely to be no specific molecular profile. These mismatch repair proficient endometrial cancers do not show the same robust response to single-agent immune checkpoint inhibition. However, pembrolizumab, in combination with lenvatinib, a multiple tyrosine kinase inhibitor, has activity and is an approved second-line therapy in this setting (90). Everolimus is an mTOR kinase inhibitor. It is believed that resistance to hormonal therapy for women with endometrial cancer may be secondary to PI3Kinase pathway activation and that mTOR kinase inhibition may help overcome endocrine resistance. In fact, the combination of everolimus and letrozole has yielded promising results in women with advanced endometrial cancer (91). The addition of metformin to everolimus and letrozole has also demonstrated promise in this setting (92). Furthermore, CDK4/6 inhibitors are being avidly explored in combination with aromatase inhibitors in ER+ endometrial cancer with some preliminary success (93).

**Figure 3:**
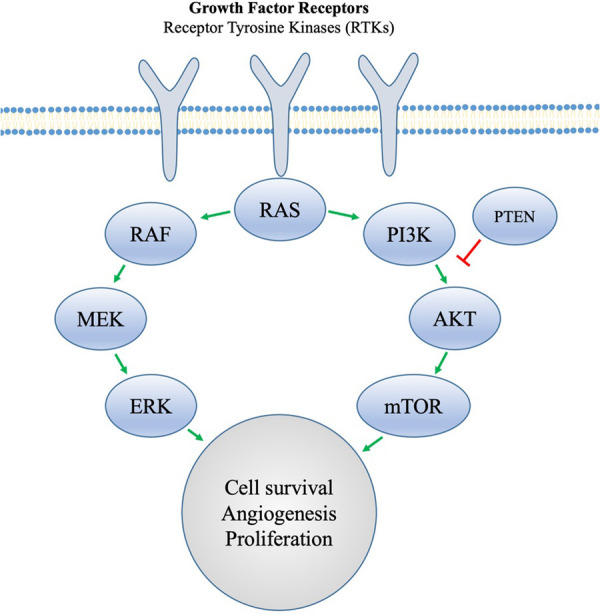
Tyrosine kinase receptors involved in angiogenesis and cell proliferation. Obesity promotes proangiogenic factors that act at the receptor level. Cancer cells that are able to evade hormonal resistance are proposed to do so through an activation of the PI3K pathway, but MTOR inhibition can be used to overcome this.

**Figure 4:**
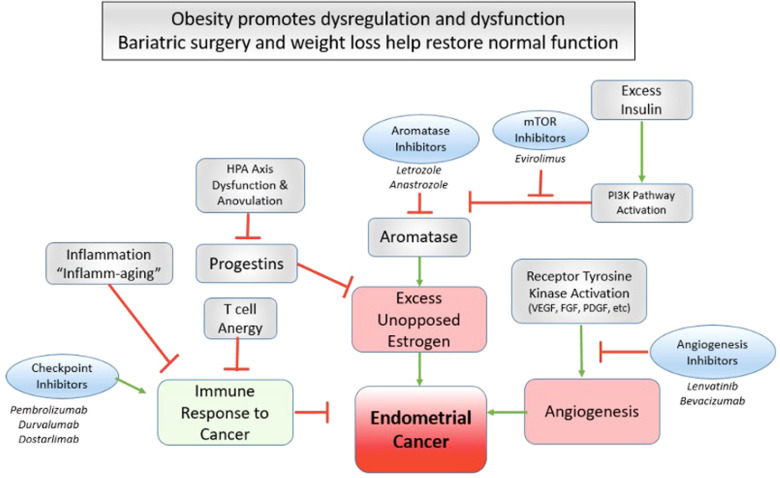
Bariatric surgery as a targeted therapy for endometrial cancer amelioration. Several novel advancements in targeted therapy for advanced endometrial cancer highlight critical pathways that may be in kind targeted effectively by bariatric surgery and successful weight loss.

Obesity is also associated with T-cell anergy and a state of “inflammaging”, which is characterized by marked chronic inflammation with an impaired immune response (94–97). Some reports suggest an enhanced immune checkpoint inhibition in patients with cancer and obesity (98), suggesting that this immune dysfunction may be uniquely targetable in this population. Bariatric surgery alters immune cell counts, lipids, and oxidation products, generally shifting from a pro-inflammatory to an anti-inflammatory phenotype (99, 100). In the series by Dyck et al. that demonstrated a 66.6% rate of regression of endometrial cancer after bariatric surgery, the authors also reported that BMI was negatively correlated with CD8 T-cell infiltration of the tumor (27). Furthermore, the addition of lenvatinib seems to enhance the efficacy of immune checkpoint inhibition, especially in the DNA mismatch repair proficient population. Lenvatinib works as a multiple kinase inhibitor acting on VEGFR 1–3 and FDGFR 1–4 among other receptors (Figure 3). One primary effect is the inhibition of angiogenesis, the markers of which are also reduced in bariatric surgery, variable with the amount of weight lost (101). Obesity and chronic states of excess insulin are implicated in carcinogenesis through the PI3Kinase pathway and excess unopposed estrogen (102, 103). Increases in estrogen and insulin seen in women with obesity lead to an activation of downstream PI3Kinase and MAPKinase pathways through a phosphorylation of AKT and ERK leading to increased cell proliferation and inhibited apoptosis (104). Successful weight loss after bariatric surgery is associated with reductions in aromatase conversion of androgens to estrogen as well as alterations in the mTOR and PI3K pathways (74, 105–108) (Figure 4).

## Conclusion

The link between obesity and endometrial cancer is clear and obesity complicates and confounds our ability to offer the best anticancer therapies to such patients. Most women with endometrial cancer will do well from a cancer standpoint but are at a very high risk of poor outcomes due to their obesity-related complications. Recent advances in anticancer therapies highlight actionable pathways such as T-cell response, anti-estrogen, and mTOR manipulation and angiogenesis; these very pathways may be therapeutically altered with bariatric surgery and weight loss. Bariatric surgery is a safe and effective treatment for obesity. We are therefore challenged to consider and explore how and where bariatric surgery might fit into the cancer treatment paradigm, particularly for early-stage patients. We may be able to harness this powerful intervention to prevent a progression of premalignant lesions to cancer, bridge patients to a better endometrial cancer surgery, effect better reproductive outcomes, prevent secondary malignancies, lead to healthier survivorship, or even produce swifter response or more durable endometrial cancer outcomes. Early metabolic surgery, particularly gastric bypass as it demonstrates the greatest risk reduction in hormonal cancers, may represent a powerful targeted therapy for patients with obesity and endometrial cancer. Well-designed, prospective, and mechanistic studies are needed to clarify the most appropriate candidates and time for bariatric surgery in this population.

## References

[1] Hales CM Carroll MD Fryar CD Ogden CL. Prevalence of obesity and severe obesity among adults: United States, 2017–2018. NCHS Data Brief. (2020) 360:1–8. PMID: https://pubmed.ncbi.nlm.nih.gov/32487284/32487284

[2] Nyberg ST Batty GD Pentti J Virtanen M Alfredsson L Fransson EI Obesity and loss of disease-free years owing to major non-communicable diseases: a multicohort study. Lancet Public Heal. (2018) 3:e490–7. 10.1016/S2468-2667(18)30139-7 PMC6178874 30177479

[3] Bianchini F Kaaks R Vainio H. Overweight, obesity, and cancer risk. Lancet Oncol. (2002) 3:565–74. 10.1016/S1470-2045(02)00849-5 12217794

[4] Calle EE Rodriguez C Walker-Thurmond K Thun MJ. Overweight, obesity, and mortality from cancer in a prospectively studied cohort of U.S. adults. N Engl J Med. (2003) 348:1625–38. 10.1056/NEJMoa021423 12711737

[5] Ciciurkaite G Moloney ME Brown RL. The incomplete medicalization of obesity: physician office visits, diagnoses, and treatments, 1996-2014. Public Health Rep. (2019) 134:141–9. 10.1177/0033354918813102 30794761 PMC6410477

[6] Carlsson L Sjöholm K Jacobson P Andersson-Assarsson JC Svensson P-A Taube M Life expectancy after bariatric surgery in the Swedish obese subjects study. N Engl J Med. (2020) 383:1535–43. 10.1056/NEJMoa2002449 33053284 PMC7580786

[7] Aminian A Kashyap SR Wolski KE Brethauer SA Kirwan JP Nissen SE Patient-reported outcomes after metabolic surgery versus medical therapy for diabetes: insights from the STAMPEDE randomized trial. Ann Surg. (2021) 274:524–32. 10.1097/SLA.0000000000005003 34132694 PMC8373787

[8] Aminian A Zajichek A Arterburn DE Wolski KE Brethauer SA Schauer PR Association of metabolic surgery with major adverse cardiovascular outcomes in patients with type 2 diabetes and obesity. JAMA. (2019) 322:1271–12. 10.1001/jama.2019.14231 31475297 PMC6724187

[9] Moussa O Ardissino M Heaton T Tang A Khan O Ziprin P Effect of bariatric surgery on long-term cardiovascular outcomes: a nationwide nested cohort study. Eur Heart J. (2020) 41:2660–7. 10.1093/eurheartj/ehaa069. 32188981

[10] Fisher DP Johnson E Haneuse S Arterburn D Coleman KJ O’Connor PJ Association between bariatric surgery and macrovascular disease outcomes in patients with type 2 diabetes and severe obesity. JAMA. (2018) 320:1570–82. 10.1001/jama.2018.14619 30326126 PMC6233803

[11] Reges O Greenland P Dicker D Leibowitz M Hoshen M Gofer I Association of bariatric surgery using laparoscopic banding, Roux-en-Y gastric bypass, or laparoscopic sleeve gastrectomy vs. usual care obesity management with all-cause mortality. JAMA. (2018) 319:279. 10.1001/jama.2017.20513 29340677 PMC5833565

[12] Eliasson B Liakopoulos V Franzén S Näslund I Svensson A-M Ottosson J Cardiovascular disease and mortality in patients with type 2 diabetes after bariatric surgery in Sweden: a nationwide, matched, observational cohort study. Lancet Diabetes Endocrinol. (2015) 3:847–54. 10.1016/S2213-8587(15)00334-4 26429401

[13] Eliassen AH Colditz GA Rosner B Willett WC Hankinson SE. Adult weight change and risk of postmenopausal breast cancer. JAMA. (2006) 296:193–201. 10.1001/jama.296.2.193 16835425

[14] Tee MC Cao Y Warnock GL Hu FB Chavarro JE. Effect of bariatric surgery on oncologic outcomes: a systematic review and meta-analysis. Surg Endosc. (2013) 27:4449–56. 10.1007/s00464-013-3127-9 23949484 PMC4018832

[15] Arnold M Pandeya N Byrnes G Renehan AG Stevens GA Ezzati M Global burden of cancer attributable to high body-mass index in 2012: a population-based study. Lancet Oncol. (2015) 16:36–46. 10.1016/S1470-2045(14)71123-4 25467404 PMC4314462

[16] Bull CJ Bell JA Murphy N Sanderson E Smith GD Timpson NJ Adiposity, metabolites, and colorectal cancer risk: Mendelian randomization study. BMC Med. (2020) 18:396. 10.1186/s12916-020-01855-9 33327948 PMC7745469

[17] Siegel RL Miller KD Fuchs HE Jemal A. Cancer statistics. CA Cancer J Clin. (2021) 71:7–33. 10.3322/caac.21654 33433946

[18] Xu M Jung X Hines OJ Eibl G Chen Y. Obesity and pancreatic cancer. Pancreas. (2018) 47:158–62. 10.1097/MPA.0000000000000974 29346216 PMC6018023

[19] Zhou B Wu D Liu H Du L Wang Y Xu J Obesity and pancreatic cancer: an update of epidemiological evidence and molecular mechanisms. Pancreatology. (2019) 19:941–50. 10.1016/j.pan.2019.08.008 31447281

[20] Kyrgiou M Kalliala I Markozannes G Gunter MJ Paraskevaidis E Gabra H Adiposity and cancer at major anatomical sites: umbrella review of the literature. Br Med J. (2017) 356:j477. 10.1136/bmj.j477 28246088 PMC5421437

[21] Choi IY Choi YJ Shin DW Han KD Jeon KH Jeong S Association between obesity and the risk of gastric cancer in premenopausal and postmenopausal women: a nationwide cohort study. J Gastroen Hepatol. (2021) 36:2834–40. 10.1111/jgh.15558 34033134

[22] Inagaki-Ohara K. Gastric leptin and tumorigenesis: beyond obesity. Int J Mol Sci. (2019) 20:2622. 10.3390/ijms20112622 PMC6600422 31141984

[23] Poorolajal J Moradi L Mohammadi Y Cheraghi Z Gohari-Ensaf F. Risk factors for stomach cancer: a systematic review and meta-analysis. Epidemiol Health. (2020) 42:e2020004. 10.4178/epih.e2020004 32023777 PMC7056944

[24] Macleod LC Hotaling JM Wright JL Davenport MT Gore JL Harper J Risk factors for renal cell carcinoma in the VITAL study. J Urol. (2013) 190:1657–61. 10.1016/j.juro.2013.04.130 23665301 PMC4420476

[25] Capitanio U Bensalah K Bex A Boorjian SA Bray F Coleman J Epidemiology of renal cell carcinoma. Eur Urol. (2018) 75:74–84. 10.1016/j.eururo.2018.08.036 30243799 PMC8397918

[26] Gild P Ehdaie B Kluth LA. Effect of obesity on bladder cancer and renal cell carcinoma incidence and survival. Curr Opin Urol. (2017) 27:409–14. 10.1097/MOU.0000000000000425 28650865

[27] Dyck L Prendeville H Raverdeau M Wilk MM Loftus RM Douglas A Suppressive effects of the obese tumor microenvironment on CD8 T cell infiltration and effector function. J Exp Med. (2022) 219:e20210042. 10.1084/jem.20210042 35103755 PMC8932531

[28] Kang C LeRoith D Gallagher EJ. Diabetes, obesity, and breast cancer. Endocrinology. (2018) 159:3801–12. 10.1210/en.2018-00574 30215698 PMC6202853

[29] Balaban S Shearer RF Lee LS Geldermalsen Mv Schreuder M Shtein HC Adipocyte lipolysis links obesity to breast cancer growth: adipocyte-derived fatty acids drive breast cancer cell proliferation and migration. Cancer Metab. (2017) 5:1. 10.1186/s40170-016-0163-7 28101337 PMC5237166

[30] Argolo DF Hudis CA Iyengar NM. The impact of obesity on breast cancer. Curr Oncol Rep. (2018) 20:47. 10.1007/s11912-018-0688-8 29644507

[31] Ceriani V Sarro G Micheletto G Giovanelli A Zakaria AS Fanchini M Long-term mortality in obese subjects undergoing malabsorptive surgery (biliopancreatic diversion and biliointestinal bypass) versus medical treatment. Int J Obesity. (2018) 43:1147–53. 10.1038/s41366-018-0244-5 30470806

[32] Gloy VL Briel M Bhatt DL Kashyap SR Schauer PR Mingrone G Bariatric surgery versus non-surgical treatment for obesity: a systematic review and meta-analysis of randomised controlled trials. Br Med J. (2013) 347:f5934. 10.1136/bmj.f5934 24149519 PMC3806364

[33] Alalwan AA Friedman J Park H Segal R Brumback BA Hartzema AG. US National trends in bariatric surgery: a decade of study. Surgery. (2021) 170:13–7. 10.1016/j.surg.2021.02.002 33714616

[34] Ghiassi S Morton JM. Safety and efficacy of bariatric and metabolic surgery. Curr Obes Rep. (2020) 9:159–64. 10.1007/s13679-020-00377-y 32253662

[35] Seeley RJ Chambers AP Sandoval DA. The role of gut adaptation in the potent effects of multiple bariatric surgeries on obesity and diabetes. Cell Metab. (2015) 21:369–78. 10.1016/j.cmet.2015.01.001 25662404 PMC4351155

[36] Ji Y Lee H Kaura S Yip J Sun H Guan L Effect of bariatric surgery on metabolic diseases and underlying mechanisms. Biomolecules. (2021) 11:1582. 10.3390/biom11111582 34827579 PMC8615605

[37] Sandoval DA. Mechanisms for the metabolic success of bariatric surgery. J Neuroendocrinol. (2019) 31:e12708. 10.1111/jne.12708 30882956 PMC9205614

[38] Schauer PR Bhatt DL Kirwan JP Wolski K Brethauer SA Navaneethan SD Bariatric surgery versus intensive medical therapy for diabetes–3-year outcomes. N Engl J Med. (2014) 370:2002–13. 10.1056/NEJMoa1401329 24679060 PMC5451259

[39] Syn NL Cummings DE Wang LZ Lin DJ Zhao JJ Loh M Association of metabolic–bariatric surgery with long-term survival in adults with and without diabetes: a one-stage meta-analysis of matched cohort and prospective controlled studies with 174 772 participants. Lancet. (2021) 397:1830–41. 10.1016/S0140-6736(21)00591-2 33965067

[40] Aminian A Brethauer SA Kirwan JP Kashyap SR Burguera B Schauer PR. How safe is metabolic/diabetes surgery? Diabetes Obes Metab. (2015) 17:198–201. 10.1111/dom.12405 25352176

[41] Arterburn D Wellman R Emiliano A Smith SR Odegaard AO Murali S Comparative effectiveness and safety of bariatric procedures for weight loss: a PCORnet cohort study. Ann Intern Med. (2018) 169:741. 10.7326/M17-2786 30383139 PMC6652193

[42] Arterburn DE Telem DA Kushner RF Courcoulas AP. Benefits and risks of bariatric surgery in adults. JAMA. (2020) 324:879–87. 10.1001/jama.2020.12567 32870301

[43] Consortium LA of BS (LABS), Flum DR Belle SH King WC Wahed AS Berk P Perioperative safety in the longitudinal assessment of bariatric surgery. N Engl J Med. (2009) 361:445–54. 10.1056/NEJMoa0901836 19641201 PMC2854565

[44] Inge TH Zeller MH Jenkins TM Helmrath M Brandt ML Michalsky MP Perioperative outcomes of adolescents undergoing bariatric surgery: the Teen–Longitudinal Assessment of Bariatric Surgery (Teen-LABS) study. JAMA Pediatr. (2014) 168:47–53. 10.1001/jamapediatrics.2013.4296 24189578 PMC4060250

[45] Kindel TL Higgins RM Lak K Gould J Kreuziger LB Mohammed A Bariatric surgery in patients with advanced heart failure: a proposed multi-disciplinary pathway for surgical care in medically complex patients. Surgery. (2021) 170:659–63. 10.1016/j.surg.2021.04.036 34052027

[46] Punchai S Hanipah ZN Sharma G Aminian A Steckner K Cywinski J Laparoscopic sleeve gastrectomy in heart failure patients with left ventricular assist device. Obes Surg. (2019) 29:1122–9. 10.1007/s11695-018-3570-8 30723879

[47] Guggino J Coumes S Wion N Reche F Arvieux C Borel A. Effectiveness and safety of bariatric surgery in patients with end-stage chronic kidney disease or kidney transplant. Obesity. (2020) 28:2290–304. 10.1002/oby.23001 33230959

[48] Spaggiari M Cocco PD Tulla K Kaylan KB Masrur MA Hassan C Simultaneous robotic kidney transplantation and bariatric surgery for morbidly obese patients with end-stage renal failure. Am J Transplant. (2021) 21:1525–34. 10.1111/ajt.16322 32976702

[49] Sjöholm K Carlsson LMS Peltonen M Taube M Response to Comment on Sjöholm et al. Association of bariatric surgery with cancer incidence in patients with obesity and diabetes: long-term results from the Swedish obese subjects study. Diabetes Care. (2022) 45:444–450. *Diabetes Care*. (2022) **45**:e73–e73. 10.2337/dci21-0065 34799430 PMC8914410

[50] Wiggins T Antonowicz SS Markar SR. Cancer risk following bariatric surgery—systematic review and meta-analysis of national population-based cohort studies. Obes Surg. (2019) 29:1031–9. 10.1007/s11695-018-3501-8 30591985

[51] Casagrande DS Rosa DD Umpierre D Sarmento RA Rodrigues CG Schaan BD. Incidence of cancer following bariatric surgery: systematic review and meta-analysis. Obes Surg. (2014) 24:1499–509. 10.1007/s11695-014-1276-0 24817500

[52] Zhang K Luo Y Dai H Deng Z. Effects of bariatric surgery on cancer risk: evidence from meta-analysis. Obes Surg. (2020) 30:1265–72. 10.1007/s11695-019-04368-4 31865552

[53] Schauer DP Feigelson HS Koebnick C Caan B Weinmann S Leonard AC Bariatric surgery and the risk of cancer in a large multisite cohort. Ann Surg. (2019) 269:95–101. 10.1097/SLA.0000000000002525 28938270 PMC6201282

[54] Rustgi VK Li Y Gupta K Minacapelli CD Bhurwal A Catalano C Bariatric surgery reduces cancer risk in adults with nonalcoholic fatty liver disease and severe obesity. Gastroenterology. (2021) 161:171–184.e10. 10.1053/j.gastro.2021.03.021 33744305

[55] Sjöholm K Carlsson LMS Svensson P-A Andersson-Assarsson JC Kristensson F Jacobson P Association of bariatric surgery with cancer incidence in patients with obesity and diabetes: long-term results from the Swedish obese subjects study. Diabetes Care. (2021) 45:444–50. 10.2337/dc21-1335 PMC8914410 34799430

[56] Pothiawala S Qureshi AA Li Y Han J. Obesity and the incidence of skin cancer in US Caucasians. Cancer Cause Control. (2012) 23:717–26. 10.1007/s10552-012-9941-x PMC3704194 22450736

[57] Taube M Peltonen M Sjöholm K Anveden Å Andersson-Assarsson JC Jacobson P Association of bariatric surgery with skin cancer incidence in adults with obesity. JAMA Dermatol. (2020) 156:38–43. 10.1001/jamadermatol.2019.3240 31664428 PMC6822159

[58] Rawla P Thandra KC Sunkara T. Pancreatic cancer and obesity: epidemiology, mechanism, and preventive strategies. Clin J Gastroenterol. (2019) 12:285–91. 10.1007/s12328-019-00953-3 30788774

[59] Almazeedi S El-Abd R Al-Khamis A Albatineh AN Al-Sabah S. Role of bariatric surgery in reducing the risk of colorectal cancer: a meta-analysis. Br J Surg. (2020) 107:348–54. 10.1002/bjs.11494 31976551

[60] Bailly L Fabre R Pradier C Iannelli A. Colorectal cancer risk following bariatric surgery in a nationwide study of French individuals with obesity. JAMA Surg. (2020) 155:395–402. 10.1001/jamasurg.2020.0089 32159744 PMC7066530

[61] Taube M Peltonen M Sjöholm K Palmqvist R Andersson-Assarsson JC Jacobson P Long-term incidence of colorectal cancer after bariatric surgery or usual care in the Swedish obese subjects study. PLoS ONE. (2021) 16:e0248550. 10.1371/journal.pone.0248550 33764991 PMC7993847

[62] Tao W Artama M Euler-Chelpin M Hull M Ljung R Lynge E Colon and rectal cancer risk after bariatric surgery in a multicountry Nordic cohort study. Int J Cancer. (2020) 147:728–35. 10.1002/ijc.32770 31797382

[63] Mackenzie H Markar SR Askari A Faiz O Hull M Purkayastha S Obesity surgery and risk of cancer. Br J Surg. (2018) 105:1650–7. 10.1002/bjs.10914 30003539

[64] Charalampakis V Tahrani AA Helmy A Gupta JK Singhal R. Polycystic ovary syndrome and endometrial hyperplasia: an overview of the role of bariatric surgery in female fertility. Eur J Obstet Gynecol Reprod Biol. (2016) 207:220–6. 10.1016/j.ejogrb.2016.10.001 27773356

[65] Balescu I Bacalbasa N Copaescu C. The effect of bariatric surgery on premalignant endometrial pathology in morbidly obese patients. Chirurgia-Bucharest. (2019) 114:704. 10.21614/chirurgia.114.6.704 31928575

[66] Argenta P Kassing M Truskinovsky A Svendsen C. Bariatric surgery and endometrial pathology in asymptomatic morbidly obese women: a prospective, pilot study. BJOG Int J Obstet Gynaecol. (2013) 120:795–800. 10.1111/1471-0528.12100 23231632

[67] Ishihara BP Farah D Fonseca MCM Nazario A. The risk of developing breast, ovarian, and endometrial cancer in obese women submitted to bariatric surgery: a meta-analysis. Surg Obes Relat Dis. (2020) 16:1596–602. 10.1016/j.soard.2020.06.008 32690459

[68] Winder AA Kularatna M MacCormick AD. Does bariatric surgery affect the incidence of breast cancer development? A systematic review. Obes Surg. (2017) 27:3014–20. 10.1007/s11695-017-2901-5 28840450

[69] Lovrics O Butt J Lee Y Lovrics P Boudreau V Anvari M The effect of bariatric surgery on breast cancer incidence and characteristics: a meta-analysis and systematic review. Am J Surg. (2021) 222:715–22. 10.1016/j.amjsurg.2021.03.016 33771341

[70] Upala S Sanguankeo A. Bariatric surgery and risk of postoperative endometrial cancer: a systematic review and meta-analysis. Surg Obes Relat Dis. (2015) 11:949–55. 10.1016/j.soard.2014.09.024 25620433

[71] Winder AA Kularatna M MacCormick AD. Does bariatric surgery affect the incidence of endometrial cancer development? A systematic review. Obes Surg. (2018) 28:1433–40. 10.1007/s11695-018-3151-x 29512036

[72] Aminian A Wilson R Al-Kurd A Tu C Milinovich A Kroh M Association of bariatric surgery with cancer risk and mortality in adults with obesity. JAMA. (2022) 3:e229009. 10.1001/jama.2022.9009 PMC9166218 35657620

[73] Zhou X Yu J Li L Gloy VL Nordmann A Tiboni M Effects of bariatric surgery on mortality, cardiovascular events, and cancer outcomes in obese patients: systematic review and meta-analysis. Obes Surg. (2016) 26:2590–601. 10.1007/s11695-016-2144-x 26992897

[74] Onstad MA Schmandt RE Lu KH. Addressing the role of obesity in endometrial cancer risk, prevention, and treatment. J Clin Oncol. (2016) 34:4225–30. 10.1200/JCO.2016.69.4638 27903150 PMC5455320

[75] Jernigan AM Maurer KA Cooper K Schauer PR Rose PG Michener CM. Referring survivors of endometrial cancer and complex atypical hyperplasia to bariatric specialists: a prospective cohort study. Am J Obstet Gynecol. (2015) 213:350.e1–e10. 10.1016/j.ajog.2015.05.015 25981846 PMC4556546

[76] Wiggins T Guidozzi N Welbourn R Ahmed AR Markar SR. Association of bariatric surgery with all-cause mortality and incidence of obesity-related disease at a population level: a systematic review and meta-analysis. PLoS Med. (2020) 17:e1003206. 10.1371/journal.pmed.1003206 32722673 PMC7386646

[77] Ward KK Shah NR Saenz CC McHale MT Alvarez EA Plaxe SC. Cardiovascular disease is the leading cause of death among endometrial cancer patients. Gynecol Oncol. (2012) 126:176–9. 10.1016/j.ygyno.2012.04.013 22507532

[78] Neff R McCann GA Carpenter KM Cohn DE Noria S Mikami D Is bariatric surgery an option for women with gynecologic cancer? Examining weight loss counseling practices and training among gynecologic oncology providers. Gynecol Oncol. (2014) 134:540–5. 10.1016/j.ygyno.2014.06.006 24933102 PMC4358811

[79] Jernigan AM Tergas AI Satin AJ Fader AN. Obesity management in gynecologic cancer survivors: provider practices and attitudes. Am J Obstet Gynecol. (2013) 208:408.e1–e8. 10.1016/j.ajog.2013.02.002 23395643

[80] Committee on Gynecologic Practice. Committee opinion No. 619. Obstet Gynecol. (2015) 125:274–8. 10.1097/01.AOG.0000459870.06491.71 25560144

[81] Mahdi H Jernigan AM Aljebori Q Lockhart D Moslemi-Kebria M. The impact of obesity on the 30-day morbidity and mortality after surgery for endometrial cancer. J Minim Invasive Gynecol. (2015) 22:94–102. 10.1016/j.jmig.2014.07.014 25064420

[82] Contreras N-A Sabadell J Verdaguer P Julià C Fernández-Montolí M-E. Fertility-sparing approaches in atypical endometrial hyperplasia and endometrial cancer patients: current evidence and future directions. Int J Mol Sci. (2022) 23:2531. 10.3390/ijms23052531 35269674 PMC8910633

[83] Malik SM Traub ML. Defining the role of bariatric surgery in polycystic ovarian syndrome patients. World J Diabetes. (2012) 3:71. 10.4239/wjd.v3.i4.71 22532886 PMC3334389

[84] Benito V López-Tomassetti E Esparza M Arencibia O Andújar M Prieto M Bariatric surgery: does it play a role in fertility-preserving treatment among obese young women with endometrial cancer? J Minim Invasive Gynecol. (2015) 22:906–9. 10.1016/j.jmig.2015.03.017 25843520

[85] Sharma N Ragupathy K. Morbidly obese patient with endometrial cancer treated by bariatric surgery to enable cancer treatment. BMJ Case Rep. (2021) 14:e243843. 10.1136/bcr-2021-243843 34301707 PMC8728343

[86] Shafa A Kumar A Torres D McKenzie TJ. Minimally invasive hysterectomy and bariatric surgery to improve endometrial cancer survivorship. Obstet Gynecol. (2019) 134:570–2. 10.1097/AOG.0000000000003403 31403586

[87] O’Malley DM Bariani GM Cassier PA Marabelle A Hansen AR Acosta ADJ Pembrolizumab in patients with microsatellite instability – high advanced endometrial cancer: results from the KEYNOTE-158 study. J Clin Oncol. (2022) 40:752–61. 10.1200/JCO.21.01874 34990208 PMC8887941

[88] FDA approves pembrolizumab for advanced endometrial carcinoma (2022). https://www.fda.gov/drugs/resources-information-approved-drugs/fda-approves-pembrolizumab-advanced-endometrial-carcinoma (accessed April 18, 2022).

[89] Wahid M Akhter N Jawed A Dar SA Mandal RK Lohani M Pembrolizumab's non-cross resistance mechanism of action successfully overthrown ipilimumab. Crit Rev Oncol Hemat. (2017) 111:1–6. 10.1016/j.critrevonc.2017.01.001 28259284

[90] Makker V Colombo N Herráez AC Santin AD Colomba E Miller DS Lenvatinib plus pembrolizumab for advanced endometrial cancer. N Engl J Med. (2022) 386:437–48. 10.1056/NEJMoa2108330 35045221 PMC11651366

[91] Slomovitz BM Jiang Y Yates MS Soliman PT Johnston T Nowakowski M Phase II study of everolimus and letrozole in patients with recurrent endometrial carcinoma. J Clin Oncol. (2015) 33:930–6. 10.1200/JCO.2014.58.3401 25624430 PMC4348638

[92] Soliman PT Westin SN Iglesias DA Fellman BM Yuan Y Zhang Q Everolimus, letrozole, and metformin in women with advanced or recurrent endometrioid endometrial cancer: a multi-center, single arm, phase II study. Clin Cancer Res. (2020) 26:581–7. 10.1158/1078-0432.CCR-19-0471 31628143 PMC7002216

[93] Mirza MR Bjørge L Marmé F Christensen RD Gil-Martin M Auranen A A randomised double-blind placebo-controlled phase II trial of palbociclib combined with letrozole (L) in patients (pts) with oestrogen receptor-positive (ER+) advanced/recurrent endometrial cancer (EC): NSGO-PALEO / ENGOT-EN3 trial. Ann Oncol. (2020) 31:S1160. 10.1016/j.annonc.2020.08.2258

[94] Porsche CE Delproposto JB Geletka L O’Rourke R Lumeng CN. Obesity results in adipose tissue T cell exhaustion. JCI Insight. (2021) 6:e139793. 10.1172/jci.insight.139793 PMC8119198 33724954

[95] Decman V Laidlaw BJ Doering TA Leng J Ertl HCJ Goldstein DR Defective CD8 T cell responses in aged mice are due to quantitative and qualitative changes in virus-specific precursors. J Immunol. (2012) 188:1933–41. 10.4049/jimmunol.1101098 22246631 PMC3320034

[96] Frasca D Blomberg BB. Inflammaging decreases adaptive and innate immune responses in mice and humans. Biogerontology. (2016) 17:7–19. 10.1007/s10522-015-9578-8 25921609 PMC4626429

[97] Castoldi A Souza Cd Câmara NOS Moraes-Vieira PM. The macrophage switch in obesity development. Front Immunol. (2016) 6:637. 10.3389/fimmu.2015.00637 26779183 PMC4700258

[98] McQuade JL Daniel CR Hess KR Mak C Wang DY Rai RR Association of body-mass index and outcomes in patients with metastatic melanoma treated with targeted therapy, immunotherapy, or chemotherapy: a retrospective, multicohort analysis. Lancet Oncol. (2018) 19:310–22. 10.1016/S1470-2045(18)30078-0 29449192 PMC5840029

[99] Jongbloed F Meijers RWJ IJzermans JNM Klaassen RA Dollé MET Berg Svd Effects of bariatric surgery on telomere length and T-cell aging. Int J Obesity. (2019) 43:2189–99. 10.1038/s41366-019-0351-y 30979972

[100] Villarreal-Calderón JR Cuéllar RX Ramos-González MR Rubio-Infante N Castillo EC Elizondo-Montemayor L Interplay between the adaptive immune system and insulin resistance in weight loss induced by bariatric surgery. Oxid Med Cell Longev. (2019) 2019:3940739. 10.1155/2019/3940739 31885787 PMC6925764

[101] Wiewiora M Mertas A Gluck M Nowowiejska-Wiewiora A Czuba Z Piecuch J. Effect of weight loss surgery on biomarkers of angiogenesis in obese patients. Obes Surg. (2020) 30:3417–25. 10.1007/s11695-020-04580-7 32307670 PMC7378109

[102] Tsugane S Inoue M. Insulin resistance and cancer: epidemiological evidence. Cancer Sci. (2010) 101:1073–9. 10.1111/j.1349-7006.2010.01521.x 20345478 PMC11159937

[103] Pollak M. The insulin and insulin-like growth factor receptor family in neoplasia: an update. Nat Rev Cancer. (2012) 12:159–69. 10.1038/nrc3215 22337149

[104] Tian W Teng F Gao J Gao C Liu G Zhang Y Estrogen and insulin synergistically promote endometrial cancer progression via crosstalk between their receptor signaling pathways. Cancer Biol Med. (2019) 16(1):55–70. 10.20892/j.issn.2095-3941.2018.0157 31119046 PMC6528450

[105] Pinhel MdS Nicoletti CF Noronha NY Oliveira Bd Cortes-Oliveira C Salgado W Mammalian target of rapamycin complex 2 signaling in obese women changes after bariatric surgery. Nutrition. (2018) 54:94–9. 10.1016/j.nut.2018.02.016 29778908

[106] Feng Y Zhong C Niu J Zhang L Zhao Y Wang W Effects of sleeve gastrectomy on lipid and energy metabolism in ZDF rats via PI3K/AKT pathway. Am J Transl Res. (2018) 10:3713–22. PMID: , PMCID: 30662621 PMC6291694

[107] Andersen CJ Murphy KE Fernandez ML. Impact of obesity and metabolic syndrome on immunity. Adv Nutr. (2016) 7:66–75. 10.3945/an.115.010207 26773015 PMC4717890

[108] Cornejo-Pareja I Clemente-Postigo M Tinahones FJ. Metabolic and endocrine consequences of bariatric surgery. Front Endocrinol. (2019) 10:626. 10.3389/fendo.2019.00626 PMC6761298 31608009

